# Supplementary material for: The genome sequence of the Reed Leopard moth, Phragmataecia castaneae Hübner, 1790 (Lepidoptera: Cossidae)
Source: Wellcome Open Res. 2025 Nov 20;10:639. [Version 1] doi: 10.12688/wellcomeopenres.25089.1 (PMC12780522; doi:10.12688/wellcomeopenres.25089.1)
Supplement: Peer review history

# Reviewer report: Reviewer response for version 1

**DOI:** 10.21956/wellcomeopenres.27659.r141774

**Published:** 2026-01-07

**Authors:** Sophie Tandonnet, Tòt Senar Serra

**Affiliations:** 1 Universitat de Barcelona Institut de Recerca de la Biodiversitat (Ringgold ID: 535620), Barcelona, Catalonia, Spain

In this genome note, the authors report the chromosome-scale assembly for the genome of *Phragmataecia castaneae*, the Reed Leopard. The assembly originates from a single male (the homogametic sex) was assembled in two haplotypes. Haplotype 1 was curated to chromosome level and contains 30 pseudo-chromosomal molecules (including the Z chromosome) and 35 additional scaffolds. This haplotype is of high quality (QV 63.9), completeness (98.4% complete BUSCOs) and highly continuous (99.8% of the genome in chromosome-scale scaffolds). The assembly is based on Pacbio HiFi long reads and scaffolded into pseudo-chromosomal molecules using Hi-C data. The same individual (ilPhrCast1) was used for the Pacbio HiFi sequencing and the Hi-C. The assembly doesn't have an annotation yet.

 The paper is straightforward and easy to follow.

 All in all, this note reports a new, high-quality and high-continuity genome assembly of a species not yet sequenced.

**Main Comments**

- It is unfortunate that the homogametic sex was sequenced instead of the heterogametic sex. The W chromosome is therefore missing and no coverage analysis can be performed to identify the Z chromosome.
- Methods, Nucleic acid extraction/HiC: The authors do not explain which part of the animal was used for the DNA extraction/Pacbio and which one was used for the HiC. For both the same sample seems to have been used and table 1 states that the whole body was used for both. Please revise this information.
-  Methods, Assembly curation: the authors state that they identified the Z chromosome by synteny (to *Zeuzera pyrina*, as stated in the report section): How conserved is the macrosynteny between Z. pyrina and *P. castanae*? Is there a clear 1 to 1 chromosomal correspondence? (there must be some difference as the chromosome number of both species doesn't seem to be the same). Maybe the authors could show the pattern of macrosynteny.
- Data availability: the authors don’t provide a link/Accession number for the RNA-seq data. 

**Minor Comments**

- Background: this section is somewhat telegraphic in style and would benefit from clearer exposition.
- Methods: The methods are well written and clear, however it would be difficult to reproduce the steps easily. For instance in "Assembly quality assessment", the authors write: "The genome is divided into chunks based on the density of BUSCO genes from the closest taxonomic lineage, and each chunk is aligned to the UniProt Reference Proteomes database with DIAMOND blastx. Sequences without hits are chunked using seqtk and aligned to the NT database with blastn ( Altschul et al. , 1990 ). The BlobToolKit suite consolidates all outputs into a blobdir for visualisation." It is unclear if all this is manual. If so, maybe add the pipeline/code to Github and reference it.

Are sufficient details of methods and materials provided to allow replication by others?

Yes

Is the rationale for creating the dataset(s) clearly described?

Yes

Are the datasets clearly presented in a useable and accessible format?

Yes

Are the protocols appropriate and is the work technically sound?

Yes

Reviewer Expertise:

Evo-Devo, sex determination, sex chromosome evolution, genomics, chromosome evolution

We confirm that we have read this submission and believe that we have an appropriate level of expertise to confirm that it is of an acceptable scientific standard.

# Reviewer report: Reviewer response for version 1

**DOI:** 10.21956/wellcomeopenres.27659.r141053

**Published:** 2025-12-30

**Authors:** Lorena Layana

**Affiliations:** 1 Institute of Science and Technology Austria (Ringgold ID: 148492), Klosterneuburg, Lower Austria, Austria

In this article, the author Acourt presents a scientific report on the genome assembly of the Lepidoptera *Phragmataecia castaneae* moth. The author assembled genomes for two haplotypes of a male *Phragmataecia castaneae* building a database of k-mer counts. Within this context, the 65 scaffolds were assembled into 30 chromosome-level scaffolds, including one Z chromosome.

 The background section it’s a nice introduction to highlight a few details of the *Phragmataecia castaneae* moth life cycle and their geographical location and spread. In the last paragraph, it is also specified that the assembly was made following the Tree of Life pipeline. Does the author refer to the pipeline used for this specific genome?, or is it a general one used for all assemblies in the Tree of Life project? I think a short sentence could be added here, or maybe including a link where the reader can have an overview of the available pipelines used by the Institute.

 The author indicates that the Z chromosome was identified according to homology to another moth species genome *Zeuzera pyrina.* But does not explicitly mention how this homology was determined. What is more, in the reference paper for the *Zeuzera pyrina* genome assembly (Boyes *et al*. 2023), I could not easily find the method by which they assigned the sex chromosome. I suggest that the author should briefly explain the method specified in Boyes *et al.* 2023, where they called it ”localised homologous pairs”.

 The quality assessment of the assembly and statistics concluded that 99.8% of the assembly (length of 590.72 Mb) was assigned to the 30 chromosome-level scaffolds, a BUSCO score of 98.4%, and a N50 of 20.82 Mb. The high quality of the sequenced data and curation allowed the author to assemble a very good genome for this Nationally rare Lepidopteran (according to Britain, Wheeler 2025).

Are sufficient details of methods and materials provided to allow replication by others?

Partly

Is the rationale for creating the dataset(s) clearly described?

Yes

Are the datasets clearly presented in a useable and accessible format?

Yes

Are the protocols appropriate and is the work technically sound?

Yes

Reviewer Expertise:

sex-chromosome evolution

I confirm that I have read this submission and believe that I have an appropriate level of expertise to confirm that it is of an acceptable scientific standard.
